# Supplementary material for: A Novel Method to Assess Motor Cortex Connectivity and Event Related Desynchronization Based on Mass Models
Source: Brain Sci. 2021 Nov 8;11(11):1479. doi: 10.3390/brainsci11111479 (PMC8615480; doi:10.3390/brainsci11111479)
Supplement: Supplementary file 1 [file brainsci-11-01479-s001.zip › brainsci-1432573-supplementary-done.pdf]

brain sciences

MDPI

A Novel Method to Assess Motor Cortex Connectivity and Event Related Desynchronization Based on Mass Models

Mauro Ursino [1*], Giulia Ricci [1], Laura Astolfi [2,3], Floriana Pichiorri [3], Manuela Petti [2,3 §] and Elisa Magosso [1 §]

[1] Department of Electrical, Electronic and Information Engineering Guglielmo Marconi, University of Bologna, Campus of Cesena, Via Dell'Università 50, I 47521 Cesena FC; giulia.ricci29@unibo.it (G.R.); elisa.magosso@unibo.it (E.M.)
[2] Department of Computer, Control and Management Engineering, Sapienza University of Rome, Via Ariosto, 25, 00185 Roma RM, Italy; laura.astolfi@uniroma1.it (L.A.); manuela.petti@uniroma1.it (M.P.)
[3] Fondazione Santa Lucia, IRCCS Via Ardeatina 306/354, Rome RM, Italy; f.pichiorri@hsantalucia.it
* Correspondence: mauro.ursino@unibo.it
§: these authors contributed equally to the present work

Mathematical Description of the Neural Mass Model

In the following, equations of a single region of interest (ROI) are first described. Then, starting from these equations a model of several interconnected ROIs is built.

Model of a single Region of Interest

The model of a single Region of Interest (ROI) consists of the feedback arrangement among four neural populations: pyramidal neurons (subscript *p*), excitatory interneurons (subscript *e*), inhibitory interneurons with slow and fast synaptic kinetics ($GABA_{A,slow}$ and $GABA_{A,fast}$, subscripts *s* and *f*, respectively). Each population receives an average postsynaptic membrane potential (say *v*) from other neural populations, and converts this membrane potential into an average density of spikes fired by the neurons (say *z*). This conversion is simulated with a static sigmoidal relationship, which reproduces the non-linearity in neuron behavior (the presence of a zone where neurons are silent (below threshold) and an upper saturation, where neurons fire at their maximal activity).

To model the dynamics in a whole ROI, the four neural populations are connected via excitatory and inhibitory synapses, according to the schema shown in the main text, in the upper panel of Fig. 1. Each synaptic kinetics is described with a second order system, but with different parameter values. We assumed three types of synapses: glutamatergic *excitatory* synapses with impulse response $h_e(t)$, assuming that synapses from pyramidal neurons and from excitatory interneurons have similar dynamics; GABAergic inhibitory synapses with *slow* dynamics (impulse response $h_s(t)$); GABAergic inhibitory synapses with *faster* dynamics (impulse response $h_f(t)$). These synapses are characterized by a gain (G_e, G_s, and G_f, respectively) and a time constant (the reciprocal of these time constants are denoted as ω_e, ω_s, and ω_f, respectively). The average number of synaptic contacts among neural populations are represented by eight parameters, C_{ij}, where the first subscript represents the target (post-synaptic) population and the second refers to the presynaptic population.

In a previous work (Ursino et al., 2010) we performed a sensitivity analysis to investigate the role of inputs to the four populations, and found that the most influential ones are those entering into pyramidal neurons and fast inhibitory interneurons. Accordingly, in this work we assume that inputs to each ROI (say *u*) target only these two neural populations (see Fig. 1 in the main text). The equations of a single ROI are written below:

Pyramidal neurons

$$\frac{dy_p(t)}{dt} = x_p(t) \quad (1)$$

$$\frac{dx_p(t)}{dt} = G_e\omega_e z_p(t) \quad -2\omega_e x_p(t) - \omega_e^2 y_p(t) \tag{2}$$

$$z_p(t) = \frac{2e_0}{1+e^{-rv_p(t)}} - e_0 \tag{3}$$

$$v_p(t) = C_{pe}y_e(t) - C_{ps}y_s(t) \quad -C_{pf}y_f(t) \tag{4}$$

Excitatory interneurons

$$\frac{dy_e(t)}{dt} = x_e(t) \tag{5}$$

$$\frac{dx_e(t)}{dt} = G_e\omega_e\left(z_e(t) + \frac{u_p(t)}{C_{pe}}\right) \quad -2\omega_e x_e(t) - \omega_e^2 y_e(t) \tag{6}$$

$$z_e(t) = \frac{2e_0}{1+e^{-rv_e(t)}} - e_0 \tag{7}$$

$$v_e(t) = C_{ep}y_p(t) \tag{8}$$

Slow inhibitory interneurons

$$\frac{dy_s(t)}{dt} = x_s(t) \tag{9}$$

$$\frac{dx_s(t)}{dt} = G_s\omega_s z_s(t) \quad -2\omega_s x_s(t) - \omega_s^2 y_s(t) \tag{10}$$

$$z_s(t) = \frac{2e_0}{1+e^{-rv_s(t)}} - e_0 \tag{11}$$

$$v_s(t) = C_{sp}y_p(t) \tag{12}$$

Fast inhibitory interneurons

$$\frac{dy_f(t)}{dt} = x_f(t) \tag{13}$$

$$\frac{dx_f(t)}{dt} = G_f\omega_f z_f(t) \quad -2\omega_f x_f(t) - \omega_f^2 y_f(t) \tag{14}$$

$$\frac{dy_l(t)}{dt} = x_l(t) \tag{15}$$

$$\frac{dx_l(t)}{dt} = G_e\omega_e u_f(t) \quad -2\omega_e x_l(t) - \omega_e^2 y_l(t) \tag{16}$$

$$z_f(t) = \frac{2e_0}{1+e^{-rv_f(t)}} - e_0 \tag{17}$$

$$v_f(t) = C_{fp}y_p(t) - C_{fs}y_s(t) \quad -C_{ff}y_f(t) + y_l(t) \tag{18}$$

The inputs to the model, $u_p(t)$ and $u_f(t)$ (Eqs. 6 and 16) represent all exogenous contributions coming from external sources (either from the environment or from other brain regions), filtered through the low-pass dynamics of the excitatory synapses (it is worth-noting that Eqs. (5)-(6) and Eqs. (15)-(16) are used to filter inputs u_p and u_f, respectively, via the dynamics of glutamatergic synapses). Indeed, a common assumption in neurophysiology is that long-range connections in the brain are always mediated via afferent

synapses from pyramidal neurons. In particular, $u_p(t)$ is the input to pyramidal cells and $u_f(t)$ the input to $GABA_{A,fast}$ interneurons. These terms will be described below.

Model of several interconnected ROIs and connectivity parameters

In order to study connectivity between regions, let us consider two ROIs (each described via Eqs. 1-18), which are interconnected through long-range connections (see the two bottom panels in Fig. 1 of the main text). The presynaptic and postsynaptic regions will be denoted with the superscript k and h, respectively. The generalization to more than two regions is trivial. Throughout the manuscript, we use the first superscript to denote the target ROI (post-synaptic) and the second superscript to denote the donor ROI (presynaptic).

To simulate connectivity, we assumed that the average spike density of pyramidal neurons of the presynaptic area (z_p^k) affects the target region via a weight factor, W_j^{hk} (where $j = p$ or f, depending on whether the synapse targets to pyramidal neurons or fast inhibitory interneurons) and a time delay, T. This is achieved by modifying the input quantities u_p^h and/or u_f^h of the target region.

Hence, we can write

$$u_j^h(t) = n_j^h(t) + W_j^{hk} z_p^k(t - T) \qquad j = p, f \tag{19}$$

where $n_j^h(t)$ represents a Gaussian white noise with a mean value m_j^h and variance $\sigma_j^2 = 9/dt$ (where dt is the integration step) and accounts for all other external inputs not included in the model.

It is worth noting that the synapses W_p^{hk} have an excitatory role on the target region h, since they directly excite pyramidal neurons (left bottom panel in Fig. 1 of the main text). Conversely, synapses W_f^{hk} , although glutamatergic in type, have an inhibitory role, via a bi-synaptic connection.

Additional model results

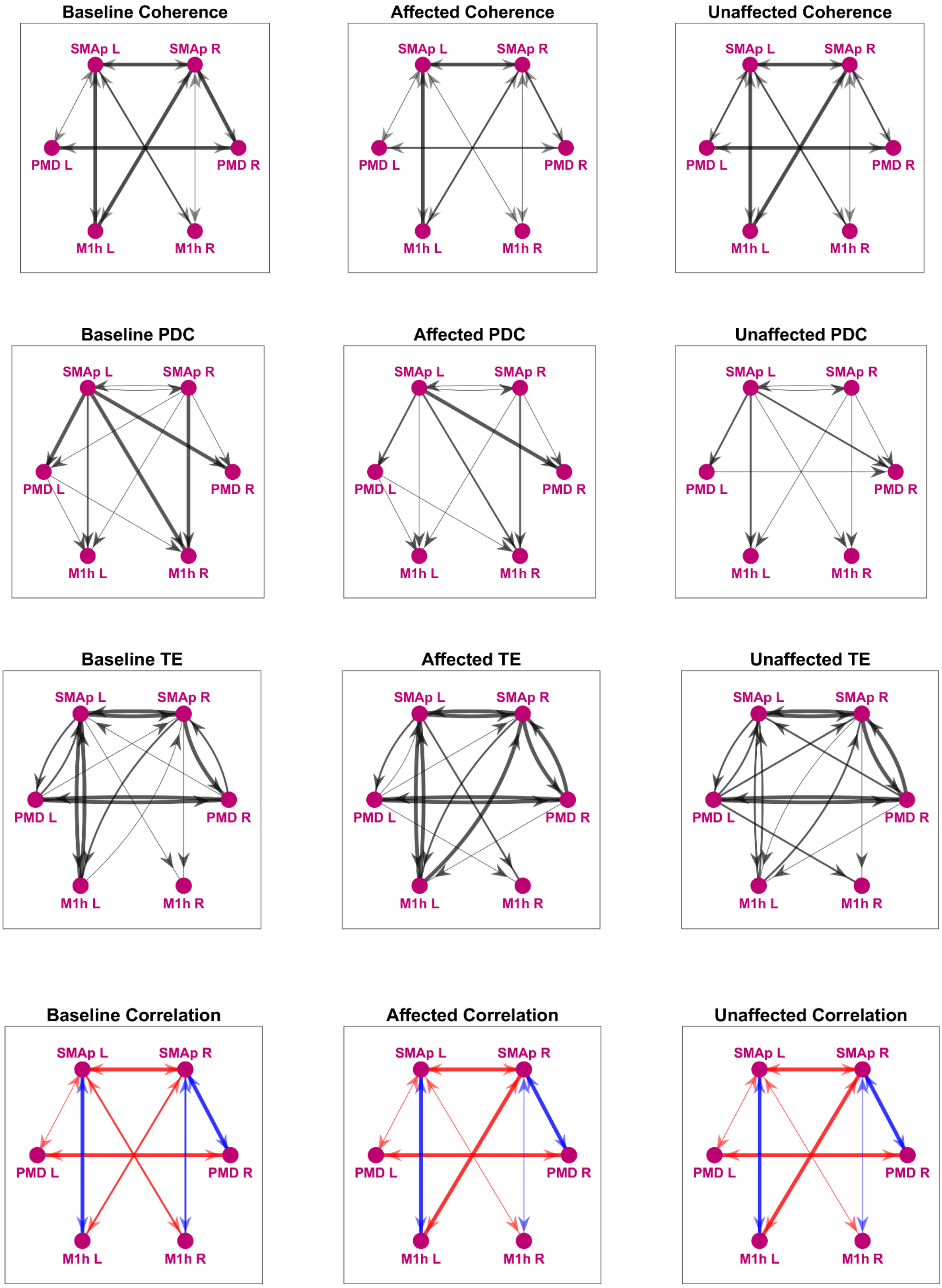

Figure S1. Connectivity networks obtained with the four different data-driven methods (Coherence, Partial Directed Coherence (PDC), Transfer Entropy (TE) and Temporal Correlation), in the three different tasks (baseline, movement of the affected hand and movement of the unaffected hand). In the temporal correlation networks, colors specify whether the bidirectional connection is *excitatory* (blue, positive correlation coefficient) or *inhibitory* (red, negative correlation coefficient).

As described in the Method section, we repeated the Step 2 of the fitting procedure to test an alternative hypothesis, i.e. assuming that the feedforward connections from each SMAp to the contralateral M1h are excitatory in type (i.e., pyramidal-pyramidal instead of pyramidal-fast inhibitory-pyramidal). The new connectivity strength resulting from the fitting are reported in Fig. S1, while the parameter values can be found in Table S1. The normalized power spectral densities of the M1h L and M1h R are shown in Fig. S2 (we do not show coherences for the sake of brevity, but the results are rather similar to those shown in Figs. 8 and 9 of the main text). As evident in Fig. S2, the model can simulate the ERD in the M1h regions rather well even assuming excitatory synapses from the contralateral SMAps. However, the fitting is worse if compared with that in Fig. 7 of the main text: in particular, during the movement of the affected hand, the model was not able to simulate an ERD in M1hR as strong as that observed experimentally (the maximum of the normalized spectrum in the model is about 0.75 compared to 0.6 in the experimental data). Furthermore, we can observe that the simulated spectra during the movement of the affected hand are shifted to lower frequencies (around 15 Hz) if compared to the experimental results (around 20 Hz). This is due to an excessive increase of the activity, close to the upper saturation of the sigmoidal relationship, which causes a decrease in the oscillation frequency.

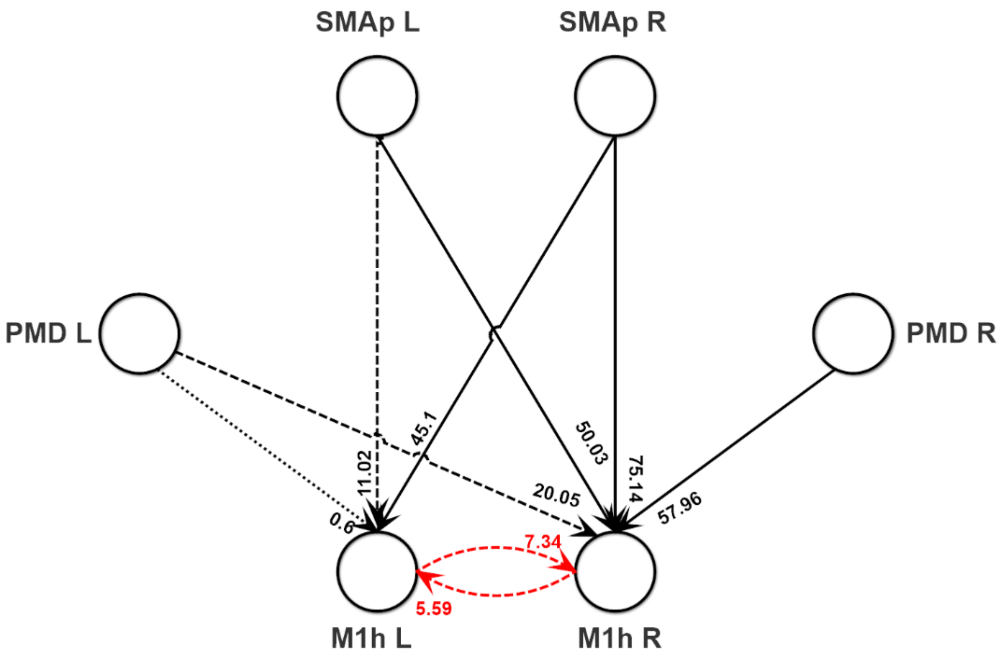

Figure S2. Connectivity strengths obtained by fitting the Neural Mass Model to the normalized power spectra and coherences for M1h L and M1h R, assuming the presence of an *excitatory* connection between each SMAp and the contralateral M1h (see Method section and Supplementary Material). Since the fitting procedure has been divided in two Steps, results of Step 1 (concerning the SMAp L, SMAp R, PMD L, PMD R) are the same as those reported in Figure 4 of the main text. Thus, the alternative fitting shown here concerns only Step 2 of the fitting procedure. Black lines denote excitatory pyramidal-pyramidal connections, whereas red lines denote inhibitory bi-synaptic connections (pyramidal-fast inhibitory-pyramidal). Continuous lines are used to denote the higher synapses, dashed lines intermediate synapses, and dotted lines the smaller synapses. All remaining synapses are set at zero. The other parameters of the fitting procedure (internal constants within each ROI) can be found in Table S1.

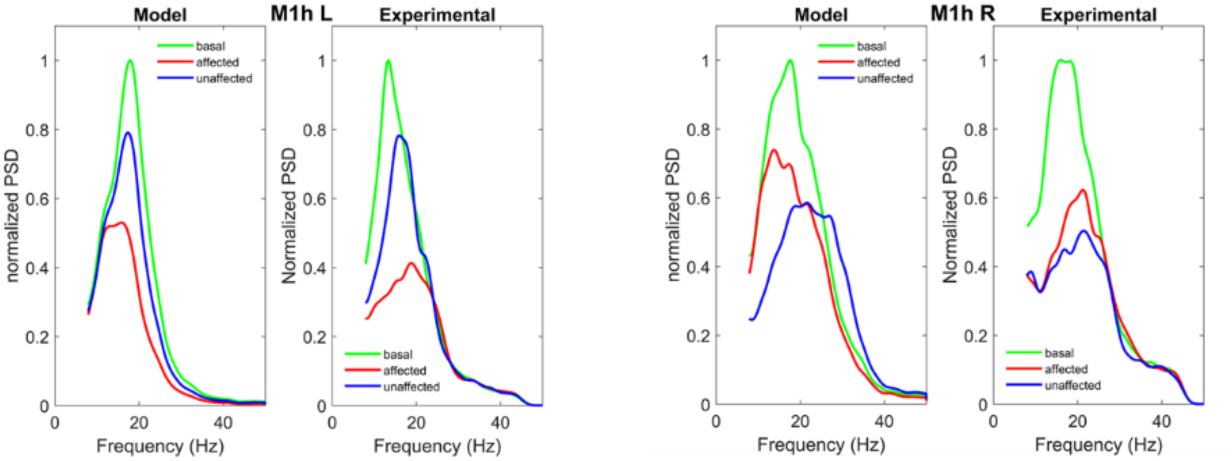

Figure S3. Normalized power spectral densities in the M1h L (left panel) and in the M1h R (right panel), obtained in basal condition (green lines) and during movement of the affected (red lines) and unaffected (blue lines) hands. In each panel, the left part represents model simulation results with optimal parameter values, and the right part the spectra computed from the experimental data. This figure differs from Figure 7 in the main text since we used the parameter values shown in Figure S2 above, i.e., assuming excitatory connections from each SMAp to the contralateral M1h.

Table S1. internal parameters estimated on ROIs M1h L, M1h R, during the alternative fitting procedure, i.e. assuming that the connections between each SMAp and the contralateral M1h are excitatory in type. It is worth noting that *ω* are the same for the two ROIs with the same value as in Table 4 of the main text.

Parameter	M1h L	M1h R	Meaning
ω_e	60.78 s^{-1}	60.78 s^{-1}	Reciprocal of a time constant
ω_s	68.24 s^{-1}	68.24 s^{-1}	"
ω_f	689.50 s^{-1}	689.50 s^{-1}	"
C_{ep}	118.08	35.53	Internal connectivity constant
C_{pe}	85.22	120.36	"
C_{sp}	5.96	63.54	"
C_{ps}	40.07	44.97	"
C_{fs}	28.07	10.87	"
C_{fp}	41.13	36.65	"
C_{pf}	97.41	142.47	"
C_{ff}	2.00	9.82	"

Copyright: © 2021 by the authors. Licensee MDPI, Basel, Switzerland. This article is an open access article distributed under the terms and conditions of the Creative Commons Attribution (CC BY) license (http://creativecommons.org/licenses/by/4.0/).

Table S2. Standard deviation of the error between model and experimental data in the range 10-30 Hz, concerning the normalized power spectral density in the six ROIs, in basal conditions and during movement of the affected and unaffected hand. These values refer to Figure 5 and 7 of the manuscript. Note that only the M1hR exhibits a significant error in basal condition, to due a shift in the peak frequency of the spectra.

	Basal	Affected	Unaffected
**SMAp L**	0.1224	0.0767	0.0739
**SMAp R**	0.0919	0.1127	0.0861
**PMD L**	0.0806	0.0743	0.0226
**PMD R**	0.0609	0.1276	0.1317

**M1h L**	0.2362	0.1979	0.0625
**M1h R**	0.5381	0.1011	0.1999

Table S3. Standard deviation of the error between model and experimental data in the range 10-30 Hz, concerning the coherences among the first four ROIs (SMAp L, SMAp R, PMD L, PMD R), in basal conditions and during movement of the affected and unaffected hand. These values refer to Figure 6 of the manuscript.

	**Basal**	**Affected**	**Unaffected**
**SMAp L - SMAp R**	0.1260	0.0971	0.0735
**PMD L - PMD R**	0.1517	0.1333	0.1601
**SMAp L - PMD L**	0.1040	0.0710	0.0938
**SMAp L - PMD R**	0.1345	0.0937	0.0894
**SMAp R - PMD L**	0.0859	0.0752	0.0592
**SMAp R - PMD R**	0.1003	0.0991	0.1218

Table S4. Standard deviation of the error between model and experimental data in the range 10-30 Hz, concerning the coherences among the last two ROIs (M1h L, M1h R) and the other four ROIs (SMAp L, SMAp R, PMD L, PMD R), in basal conditions and during movement of the affected and unaffected hand. These values refer to Figures 8 and 9 of the manuscript.

	**Basal**	**Affected**	**Unaffected**
**M1h L - M1h R**	0.1301	0.1011	0.0907
**M1h L - SMAp L**	0.1652	0.1451	0.1072
**M1h L - SMAp R**	0.1056	0.1166	0.0905
**M1h L - PMD L**	0.1199	0.0964	0.0801
**M1h L - PMD R**	0.1391	0.0947	0.1229
**M1h R - SMAp L**	0.0924	0.0981	0.0754
**M1h R - SMAp R**	0.1683	0.1536	0.1314
**M1h R - PMD L**	0.0946	0.0748	0.0409
**M1h R- PMD R**	0.0854	0.0575	0.0550
